# How government insurance coverage changed the utilization and affordability of expensive targeted anti-cancer medicines in China: an interrupted time-series study

**DOI:** 10.7189/jogh.09.020603

**Published:** 2019-12

**Authors:** Yifan Diao, Jie Qian, Yang Liu, Yanping Zhou, Yan Wang, Hong Ma, Xiaoyan Wang, Ren Luo, Anita Wagner, Jing Sun, Yuanli Liu

**Affiliations:** 1 School of Public Health, Chinese Academy of Medical Sciences & Peking Union Medical College, Beijing, China; 2 Department of Internal Oncology, Peking Union Medical College Hospital, Beijing, China; 3 Cancer Hospital, Chinese Academy of Medical Sciences, Beijing, China; 4 North Head Strategic Communications Healthcare Research, Beijing, China; 5 IQVIA (IMS Health and Quintiles) Institute for Healthcare Informatics, Beijing, China; 6 Department of Population Medicine, Harvard Medical School and Harvard Pilgrim Health Care Institute, Boston, Massachusetts, USA; **Background** Evidence is lacking about the impact of emerging government health insurance coverage inclusion on patient utilization and affordability of expensive anti-cancer medicines and insurance sustainability in China.

## Abstract

**Methods:**

Using an interrupted time series design, we conducted segmented regression analyses of utilization changes of targeted anti-cancer medicines covered by the provincial government health insurance program during 2013 to 2016 in 69 hospitals with more than 100 beds in Hangzhou, the capital city of Zhejiang province of China. The WHO/Health Action International Project on Medicine Prices and Availability methodology was used to measure patient affordability of the study medicines.

**Results:**

In March 2015, the utilization of all study medicines increased by 15.58 (95% CI = 3.86, 27.30, *P* = 0.01) to 439.14 standard units (95% CI = 311.79, 566.49, *P* < 0.001). Before covered by government health insurance, the estimated out-of-pocket payment by patient ranged from 3.0 to 13.1 times of the provincial average disposable annual income per capita for urban residents, and 6.2 to 27.3 times for rural residents. Such payments were reduced to 0.6 to 2.1 times for urban residents and 1.8 to 4.4 times for rural population after government health insurance coverage inclusion. During 2015 to 2016, the per capita contribution to Hangzhou catastrophic health insurance program was CNY15 (US$ 2.3), and the reimbursement rate was 70% in Hangzhou city. The cumulative total insurance expenses on six study targeted anticancer medicines accounted for an estimated 53% of the total amount of premiums of the government catastrophic health insurance fund. Sensitivity analyses indicated that this proportion would have changed to 46%, 61% and 69% when changing the per capita contribution to CNY25 (US$ 3.8) and CNY40 (US$ 6.2), and changing the insurance reimbursement rate to 60%, 80 and 90%.

**Conclusion:**

Government health insurance coverage inclusion significantly increased utilization of the expensive targeted anti-cancer medicines, and improved patient affordability. However, the financial burden of patients is still high, especially for the rural low-income population. Rising utilization and expenditures call for careful monitoring of anti-cancer medicines use, and for strategies to decrease prices to facilitate medicines access and keep the insurance system sustainable.

Cancer incidence and mortality have been increasing in China, making cancer the leading cause of death since 2010 and a major public health problem in the country [1]. New targeted therapeutics have brought hope to many cancer patients and improved their quality of life. However, skyrocketing prices make government insurance coverage inclusion of these medicines intricately intertwined with socio-political issues in the face of emotion and public pressure [2]. Scientific evidence on use and spending of medicines is essential for an efficient and sustainable health insurance program. China has just begun to include targeted anti-cancer medicines into its government health insurance system following price negotiations at the national level in 2016. Some areas have already included these medicines into their local health insurance programs. As detailed policies are determined by individual local health insurance programs, benefit packages vary with respect to coverage inclusion of these expensive medicines by local fundraising levels and different financial risk protection capacities. There is little evidence available for decision makers about changes in utilization of and spending on these medicines after they are included in government health insurance reimbursement programs. Little is known about the effects of reimbursement on patients’ financial burden and the sustainability of the insurance programs.

To support government health insurance policy design and evaluation, we presented the evidence on cancer medicine access and spending in Hangzhou city, the capital city of Zhejiang province. In recent years, the crude incidence of malignant tumors in Zhejiang province has been higher than the national average [3,4]. Zhejiang is one of few provinces that included expensive medicines into the benefit package of the local catastrophic health insurance program in 2015, and hospitals procure the medicines at the provincially negotiated prices [5]. We analyzed the impact of insurance coverage inclusion of the targeted anti-cancer medicines on utilization of these medicines in Hangzhou from January 2013 through December 2016. We also studied patient affordability of these medicines and the impact of insurance coverage inclusion of these medicines on insurance spending.

## METHODS

### Study design

We combined quantitative and qualitative research methods to estimate changes in utilization, patient affordability and insurance spending of the study medicines.

#### Utilization

To assess the utilization, we analyzed the monthly sales data in volume of the study medicines. Standard Unit as defined by IQVIA was used to quantify the utilization. Using longitudinal sales data, we assessed utilization changes over January 2013 to December 2016 of the targeted anti-cancer medicines before and after the insurance coverage inclusion.

#### Patient affordability

To measure the patient affordability, we adopted the adjusted WHO/Health Action International (WHO/HAI) Project on Medicine Prices and Availability methodology of Measuring medicine prices, availability, affordability and price components [6]. The number of daily wage of the lowest-paid unskilled government worker to purchase selected courses of treatment for common acute and chronic conditions was adjusted by calculating the number of average per capita disposable annual income needed to pay for the out-of-pocket (OOP) expenditure of the study medicines by patient. The calculation was based on the standard treatment guideline of respective regimes for a duration of either one year for imatinib or the median survival time of other study medicines. To assess changes in patient’s affordability of the study medicines, we analyzed the number of average per capita disposable annual income needed to cover the OOP expenditure for the study medicines by patients both eligible and not eligible for pharmaceutical companies’ patient assistance program (the program is only open to the low-income population as defined by the local government) with the following scenarios: (1) Before insurance coverage inclusion, urban and rural patients who were not eligible for the patient assistance program had to pay the study medicines totally OOP; (2) Before insurance coverage inclusion, urban and rural patients who were eligible for the patient assistance program paid for a defined number of initial doses. The patient assistance program paid another defined number of doses. Patients had to pay for the remaining number of doses if the median survival time is longer, and additional doses were needed. The national lowest provincial pooled procurement price was applied to calculate medication expenses [3]. After insurance coverage inclusion, local government negotiation resulted price reduction, and an enhanced patient assistance program was introduced, which enabled every insured patient eligible for the patient assistance program. And the number of doses paid by patient assistance program increased. With the insurance coverage inclusion, after a deductible of CNY20 000 (US$ 3077), 70% of the total amount of patient’s payment as described in the second scenario was paid by the government health insurance, and patients paid 30%. The negotiated price was applied to calculate medication expenses.

#### Financial impact on insurance spending

To assess the financial impact on insurance spending, we estimated the proportion of insurance spending on the study medicines as a proportion to the total amount of funds collected by the Hangzhou catastrophic health insurance program during 2015 and 2016.

We first summed the sales of the study medicines in value during 2015 and 2016, and calculated the insurance spending on these medicines with the insurance reimbursement rate of 70%. We had the insurance spending divided by the total amount of funds collected by Hangzhou catastrophic health insurance program, which was calculated based on CNY15 (US$ 2.3) per capita contribution per year during 2015 and 2016.

### Medicines, population, setting and data source

The study medicines were six targeted anti-cancer medicines (cetuximab, gefitinib, imatinib, trastuzumab, and rituximab [in two strengths]) included in the benefit package of Hangzhou’s catastrophic health insurance program in 2015. We analyzed longitudinal monthly utilization (as number of standard units procured) of these medicines in all 69 hospitals with more than 100 beds in Hangzhou between January 2013 and December 2016. Data were extracted from the IQVIA Chinese Hospital Pharmaceutical Audit (CHPA) system and included ATC code, generic name, brand name, manufacturer, dosage form, strength, package size, and monthly procurement volumes. The population size of Hangzhou and the annual per capita contribution by each citizen to the Hangzhou catastrophic health insurance program in 2015 and 2016 were obtained from Hangzhou Statistics Bureau and Hangzhou Municipal Human Resource and Social Security Bureau [7-9]. Negotiated medicines prices and information on the benefit package of the catastrophic health insurance program of Hangzhou were obtained from the program’s website and through interviews of key informants of the insurance program [10,11]. Medicines prices before insurance inclusion were obtained from the national database of the provincial pooled procurement of medicines [12]. The per capita disposable incomes of urban and rural residents of Zhejiang province in 2014 and 2016 were obtained from the Statistics Bureau of Zhejiang Province [13]. Pharmaceutical company patient assistance program information was obtained from the official websites of the respective charity foundations that cooperated with the pharmaceutical companies [14-17]. Necessary clinical information on the study medicines, including the median survival times of medications for the targeted indications were obtained from the published peer reviewed literature and the official website of the National Database of Registered Medicines [18-26].

### Statistical and sensitivity analyses

Conducting segmented linear regression analyses of the longitudinal time series consumption data of the study medicines, we assessed changes in levels and trends after the insurance inclusion compared to before inclusion. As the policy was issued in February 2015, we regarded March 2015 as the time for the policy implementation to take effect. Segmented linear regression divided the time series into pre-March and post-March 2015 segments. The Durbin-Waston test was used to evaluate autocorrelation of errors. The analysis was performed with SPSS 21.0 with the significant level set at 0.05 for a two-sided test.

We also conducted sensitivity analyses to assess the financial impact of the medicines inclusion on insurance spending. We assumed that 69 hospitals were the major source of utilization of the study medicines in Hangzhou, and all patients who consumed the study medicines in the 69 hospitals were covered by the Hangzhou catastrophic health insurance program. The actual insurance contribution in Hangzhou was CNY15 (US$ 2.3) per year per capita, and the actual insurance reimbursement rate was 70% in 2015 and 2016 [9]. Intended to cover all possible scenarios of insurance fund collection and spending in the study area, we assumed that the insurance reimbursement rates of the study medicines to be changed to 60%, 80% and 90%, and the annual per capita contribution to the Hangzhou catastrophic health insurance program to be increased to CNY25 (US$ 3.8) and CNY40 (US$ 6.2). Different proportions of insurance expending for the study medicines to the total amount of insurance fund collected were calculated under various conditions.

## RESULTS

### Changes of utilization of the study medicines

**Figure 1:**
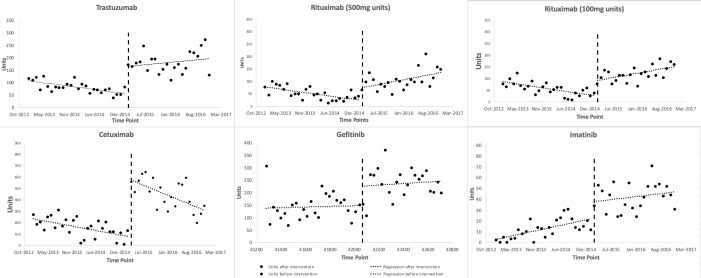
Regression results of the monthly utilizations of the study medicines before and after insurance inclusion.

**Figure 1** shows scatter plots of the observed monthly utilization of the study medicines. March 2015 was regarded as the onset time of the insurance inclusion, which divided the time series into two parts, ie, before and after the insurance coverage inclusion of the study medicines. The two lines before and after March 2015 were plotted based on the segmented linear regression model. The regression results (**Table 1**) show that the monthly utilization of each study medicines increased suddenly in March 2015, with a range of level changes between 15.58 (95% CI = 3.86, 27.30, *P* = 0.01, for imatinib) and 439.14 (95% CI = 311.79, 566.49, *P* < 0.001, for cetuximab) standard units. Prior to March 2015, the baseline trends of the monthly utilization of gefitinib increased (*P* = 0.79), and that of imatinib increased significantly (*P* = 0.005), and those of the other medicines decreased significantly (*P* = 0.018, *P* = 0.005, *P* < 0.001, *P* = 0.029). After March 2015, the monthly utilization trends of rituximab (500mg/100mg) increased significantly (*P* = 0.003, *P* < 0.001), while that of cetuximab decreased (*P* = 0.037), and those of the other medicines steadily increased (*P* = 0.17, *P* = 0.63, *P* = 0.21). The trends of trastuzumab and rituximab (500mg/100mg) changed from significant decreases during baseline to significant increases after the inclusion (*P* = 0.012, *P* < 0.001, *P* < 0.001), while the trend changes of the other medicines after the inclusion were not statistically significant (*P* = 0.75, *P* = 0.82, *P* = 0.43).

**Table 1:** Regression estimates of monthly utilization changes of the study medicines after insurance coverage inclusion, compared with before insurance coverage inclusion*

Study medicines	Intercept	Baseline trend (95% CI, *P-* value)	Level change (95% CI, *P-*value)	Trend change (95% CI, *P-*value)	Post inclusion trend (95% CI, *P-*value)
Trastuzumab	**109.52**	**-2.02** (-3.67 to 0.37, 0.018)	**106.93** (70.14 to 143.71, <0.001)	**3.50** (0.82 to 6.19, 0.012)	1.48 (-0.64 to 3.60, 0.17)
Rituximab (500mg)	**79.56**	**-2.08** (-3.52 to -0.65, 0.005)	**46.50** (14.43 to 78.58, 0.005)	**5.18** (3.14 to 7.21, <0.001)	**2.92** (1.07 to 4.77, 0.003)
Rituximab (100mg)	**89.48**	**-2.36** (-3.61 to -1.11, <0.001)	**61.79** (33.87 to 89.70, <0.001)	**5.01** (2.66 to 7.35, <0.001)	**2.82** (1.21 to 4.42, <0.001)
Cetuximab	**242.51**	**-6.39** (-12.1 to -0.68, 0.029)	**439.14** (311.79 to 566.49, <0.001)	-1.45 (-10.45 to 7.85, 0.754)	**-7.84** (-15.18 to -0.51, 0.037)
Gefitinib	**137.71**	0.39 (-2.59 to 3.37, 0.79)	**77.89** (11.45 to 144.34, 0.023)	0.54 (-4.31 to 5.39, 0.82)	0.93 (-2.90 to 4.76, 0.63)
Imatinib	**1.99**	**0.76** (0.24 to 1.29, 0.005)	**15.58** (3.86 to 27.30, 0.01)	-0.34 (-1.20 to 0.52, 0.43)	0.42 (-0.25 to 1.10, 0.21)

*Bold means statistically significant change.

### Changes of patient’s affordability

**Table 2** shows that patient’s affordability of the study medicines decreased from a range of 3.0 to 13.1 years of the average annual income for urban patients, and 6.2 to 27.3 years for rural patients in 2014. These patients paid the study medicines entirely OOP. For those who were eligible for the patient assistance program, 1.5 to 6.4 years of the average annual income for urban patients and 3.1 to 13.4 years for rural patients would need to be paid for the OOP expenditures of respective study medicines. After the insurance coverage inclusion, patient’s affordability reduced to a range of 0.6 to 2.1 years of the average annual income for urban patients and 1.8 to 4.4 years for rural patients to pay respective study medicines OOP.

**Table 2:** Patient affordability of the study medicines for a standard course of treatment before and after the insurance coverage inclusion

**Study medicines**	**Indication, treatment course based on median survival time, usage & dosage**	National lowest pooled procurement price (CNY/US$)*	**Before insurance inclusion**					**After insurance inclusion**		
			OOP of patient(non-eligible for patient assistance program (CNY/US$)*	Patient affordability**	Pharmaceutical company patient assistance program and price (CNY/US$)*	OOP of patient(eligible for patient assistance program, (CNY/US$)*	**Patient affordability†**	Pharmaceutical company patient assistance program and price(CNYUS$)*	OOP of patient(everyone is eligible for patient assistance program, (CNY/US$)*	**Patient affordability‡**
Trastuzumab	**Breast cancer:** 18.5 mo 21 vials 440 mg:20 ml/vial	21 613/3325	453 873/69 827	U:11.2, R:23.4	6 vials paid by patient assistance program after initial 6vials paid by patient, 10 807/1663 per vial	259 365/39 902	**U:6.4, R:13.4**	8 vials paid by patient assistance program after initial 6 vials co-paid by patient and insurance, 19 000/2923 per via	100 400/15 446	**U:2.1, R:4.4**
	**Gastric cancer:** 13.8 mo 16 vials 440mg:20ml/vial		345 808/53 201	U:8.6, R:17.8		216 130/33 251	U:5.4, R:11.2	Company donates 7 vials after payment of 5 vials, 19 000/vial	80 400/12 563	**U:1.7, R:3.5**
Rituximab	**Lymphoma:** 8 weeks, 51 bottles, 100 mg:10ml/bottle	3416/526	174 216/26 802	U:4.3, R:9.0	No patient assistance program	/	/	Company donates 4 mo after payment of 4 mo, 3400/623 per bottle(100mg)since 1, June 2015	81 360/12 517	U:1.7, R:3.5
Cetuximab	**Colon cancer**: 8 mo, 139 bottles, 20ml:100mg/bottle	3805/475	528 895/81 368	**U:13.1, R:27.3**	5 mo paid by patient assistance program after initial 3 mo paid by patient, 1 427/220 per bottle	199 336/30 667	U:4.9, R:10.3	Free after payment of 3 mo, 3 800/585 per bottle	91 230/14 035	U:1.9, R:4.0
Gefitinib	**Lung cancer:** 10 mo, 30 boxes, 250 mg*10	4000/615	120 000/18 462	**U:3.0, R:6.2**	Free after initial 5 mo paid by patient, 2000/308 per box	60 000/9231	**U:1.5, R:3.1**	Free after payment of 4 mo, 4000/ 615 per box	31 200/4800	**U:0.7, R:1.4**
Imatinib	**Leukemia:** 1year, 25 boxes, 0.1 g*60 tab/box	10 800/1662	270 000/41 538	U:6.7, R:14.0	6 mo paid by patient assistance program after initial 6 mo paid by patient, 5 400/831 per box	135 000/20 769	U:3.3, R:7.0	Company donates 9 mo after payment of 3 mo, 10 000/ 1 538 per box	37 000/5692	U:0.8, R:1.6

U – urban patients, R – rural patients, OOP – out-of-pocket, mo – months

*US$ 1 = CNY6.5.

†Annual per capita disposable income of urban and rural residents in Zhejiang Province in 2014 (CNY/US$ 40 393/6 214 and 19 373/2 980).

‡Annual per capita disposable income of urban and rural residents in Zhejiang Province in 2016 (CNY/US$ 47 237/7 267 and 22 866/3 518).

### Changes of the financial impact on insurance spending

IQVIA CHPA data showed that the total value of the study medicines used in 69 hospitals of Hangzhou with >100 beds during 2015 and 2016 was CNY202 232 763 (US$ 31 112 733). When the overall insurance reimbursement rate for the medicines was set at 60%, the insurance spending on the study medicines during 2015 and 2016 was CNY121 339 658 (US$ 18 667 640). Following the contribution standard set by the Hangzhou catastrophic health insurance program, CNY15 (US$ 2.3) should have been contributed per person per year (before 2017) [9], and according to the total population of Hangzhou (8.71 million in 2015 and 8.98 million in 2016) [7,8], the total amount of funds collected by the Hangzhou catastrophic health insurance program should have been CNY2.65 billion (US$ 407.7 million). Based on these assumptions, in the first two years of the insurance inclusion, 46% of the total funds collected by Hangzhou catastrophic health insurance program during 2015 and 2016 would have been spent on the six targeted anti-cancer medicines. Sensitivity analyses (**Table 3**) show that when changing the insurance reimbursement rate from 70% to 60%, 80% and 90%, the proportion of funds allocated to these six anti-cancer medicines would have been between 46%-69%. If setting the per capita contribution level at CNY25 (US$ 3.8), the proportions of allocated funds is between of 27% and 41%. The proportions decrease to between 17% and 26% with the per capita contribution raised to CNY40 (US$ 6.2).

**Table 3:** Financial impact of the inclusion of six targeted anti-cancer medicines in the Hangzhou catastrophic health insurance program, with sensitivity analysis results*

Total expenses on six study targeted anti-cancer medicines 2015-2016 (CNY/US$)		Total insurance spending on six study targeted anti-cancer medicines 2015-2016 (CNY/US$)			
**Reimbursement rate**			
		60%	70%	80%	90%
202 232 763/31 112 733		121 339 658/18 667 640	141 562 934/21 778 913	161 786 210/24 890 186	182 009 487/28 001 460
**Per capita contribution to Hangzhou catastrophic health insurance program (CNY/US$)**	**Total amount of funds collected by Hangzhou catastrophic health insurance program 2015-2016 (100 000 CNY/US$)**	**Proportion of insurance spending on six study targeted anti-cancer medicines to total amount of funds collected by Hangzhou catastrophic health insurance program 2015-2016 (%)**			
15/2.3	2653.5/408.2	46	53	61	69
25/3.8	4422.5/680.4	27	32	37	41
40/6.2	7076.0/1 089	17	20	23	26

*US$ 1 = CNY6.5.

## DISCUSSION

### Utilization of the study medicines significantly increased

Health insurance reimbursement in March 2015 abruptly and significantly increased the utilization of the expensive targeted anti-cancer medicines. This effect continued throughout the observation time frame. The monthly utilization trend of cetuximab decreased significantly after the insurance inclusion. This decrease may be associated with the limitation of indications by the insurance program. The only indication of cetuximab covered by the insurance is irinotecan-resistant metastatic colon cancer, which requires genetic testing before starting the medication. Although the incidence of colorectal cancer is high in Zhejiang province, there was another new targeted therapy, bevacizumab, marketed in China with the same indication, colorectal cancer. Treatment with bevacizumab does not require genetic testing. Both medicines had similar patient assistance program pre-conditions in 2015 (initial doses of treatment to be paid by patients for 3 months and 4 months, respectively), making the economic burden of the two medicines almost the same. However, if the cost of genetic testing for cetuximab is added, the overall treatment cost of cetuximab will be higher than that of bevacizumab. In addition, considering that bevacizumab is used in the earlier stage of colorectal cancer treatment, it would not be easy to change medicines during treatment if the treatment is effective. Insurance coverage inclusion might attract some patients for a short while, but indication limitation and gene testing might divert some patients from taking cetuximab to bevacizumab.

### Other factors might affect utilization of the study medicines

Imatinib was the only study medicines with a significantly increasing trend in monthly consumption before the insurance inclusion. Imatinib has dramatically changed the life expectancy of chronic myelocytic leukemia patients and is used long term, possibly accounting for continually increasing trends. Further, many patients sought overseas shopping services for medicines with large price gaps between China and other countries [27]. In 2014, Lu Yong, a leukemia patient who helped other patients buy imatinib from India, was sentenced for violating national laws of selling non-registered medicines [27]. This might have discouraged patients from seeking overseas shopping services for imatinib (which would not be captured in our data) and contribute to increasing imatinib sales which are measured by our data. Rituximab, cetuximab, and trastuzumab had significantly decreasing utilization trends before insurance coverage inclusion. One possible explanation might be an anticipatory effect that, new patients and their prescribers may have waited to start the medicines until the insurance coverage inclusion was implemented resulting in a backlog of patients needing the medicines and explaining the sudden level changes in March 2015.

### Patient’s affordability improved

The financial burden of the study medicines was tremendous and not affordable at all for Chinese patients in 2014. Although the pharmaceutical companies’ patient assistance programs may have relieved the burden to some extent, the medications were still unaffordable for patients in urban areas who were estimated to spend 1.5 to 6.4 times their annual income for a standard course of treatment. The affordability problem is even more serious for the low income population, especially for those low-income patients in rural areas, even after coverage inclusion by the government health insurance program..

### Local generics to substitute the originators

Among the study medicines, imatinib was the only one with expired patent protection in China at the time of the study. We sought to analyze the utilization of local generics of imatinib together with that of the branded originator. We were surprised to find that there was nearly no use of the local generics of imatinib in the hospitals of Hangzhou, although prices of generic products were much lower than those of the originator. We learned that generics of the study medicines had not yet completed the required quality and clinical efficacy validation. As soon as generics complete this validation, they should be favored in the next rounds of price negotiations and reimbursement inclusion, and are expected to break the monopoly of the originator. This is especially the case since the Ministry of Human Resources and Social Security suggested that generics should be promptly included by the health insurance program [28].

### Implications for the government insurance programs

One important finding of this study is the equity issue of the government inclusion policy of the expensive anti-cancer medicines. It calls for continued strengthening of financial risk protection for the low income population to avoid excluding them from accessing cancer medicines. It is good to see that equal access has been addressed by the government recently through the Health Poverty Alleviation Initiative [29], which is intended to build a health safety net for the patients in financial hardship. Through which, health expenditures of the patients in financial hardship beyond a fixed amount will be covered by the initiative. Resources are allocated by the central and locally governments specifically and secured. Effect of this initiative needs further evaluation.

Inclusion of the six targeted anti-cancer medicines in the benefit package led to an estimated spending of around 46% of the total amount of funds collected by the Hangzhou catastrophic health insurance program during 2015 and 2016. This spending did not include other medical expenditures such as diagnostic tests. In addition, 60% is the lowest reimbursement rate of health insurance programs, some other areas adopt up to a rate of 90%, which will lead to an estimated 69% of insurance funds spent on six targeted anti-cancer medicines. Even though when the per capita fund collection level is increased to CNY25 (US$ 3.8) and CNY40 (US$ 6.2), the annual total fund will almost triple, it is likely that future expenditures for these medications will grow rapidly given potentially increased consumption and increasing incidence of cancers in Hangzhou. Although the utilization could also be associated with factors like income, employment, residence, access to health facility of individual patients, as we measured the utilization in the setting of Hangzhou city, and targeted the population of the city, not at individual level, the above factors may not change significantly across the study time. Furthermore, the above calculations are for six medicines only. There were 15 expensive medicines included in the 2015 reimbursement list, and another 21 expensive medicines were included in 2018 [10]. These important coverage expansions imply rising insurance expenditures in the future and call for careful monitoring of use of these expensive medicines and further price reduction through price negotiations and generic competition.

In addition, most of the insurance-covered indications for the medicines require genetic testing to identify patients who could benefit from the medicines. Considering that most health insurance programs (including Hangzhou) do not cover genetic test expenditures, OOP spending on tests will further raise the financial burden of patients, especially of the patients in financial hardship. This will further block access of the low income population. It is good that some regional insurance programs have been exploring inclusion of genetic testing into their benefit packages and bundling tests for expensive targeted medicines in price negotiations. In addition, barrier of access to genetic test may lead to non-adherence to clinical guidelines of targeted anti-cancer medication without the guidance of genetic tests, which may lead to potentially ineffective use [30]. In view of the high expenses of these medicines, promoting precision medication following the clinical guidelines will have great implications for an efficient and sustainable development of the government insurance system in the future.

### Limitations

To our knowledge, this is the first study with a robust interrupted time series design to measure the utilization and affordability of the government health insurance inclusion of expensive anti-cancer medicines in China. It is based on the following assumptions. First, considering that hospitals tend to optimize the inventory of pharmaceuticals to reduce management costs, monthly medicines purchases in IQVIA data reflect the monthly consumption. Second, hospital pharmacies are still the main source of medicines for patients in China. However, since Hangzhou’s catastrophic health insurance program allows reimbursement of medicines dispensed in designated retail pharmacies, and allows for direct to patient (DTP) services [31] especially of oral targeted products, sales through and spending in retail settings should be included in future studies. Third, patient’s affordability was calculated based on a standard treatment and median survival time, and we assumed that patients are eligible for the most optimized patient assistance program. In reality, the actual benefits of patients vary extensively. Our results may thus overestimate the effect of patient assistance programs on reducing the financial burden of patients. Fourthly, we assumed that all residents of Hangzhou city enrolled in the basic health insurance and the catastrophic health insurance programs. If this is not the case, we would underestimate the impact on the insurance fund of reimbursing for the six medicines. Consumption data were collected from hospitals of Hangzhou, which treat both local and external patients. We were not able to exclude external patients with insurance policies of other provinces. This inclusion of other patients would only bias our estimates of medicine utilization changes associated with Hangzhou’s coverage policies, if the proportion of external patients receiving the study medicines in Hangzhou would have changed at the same time as Hangzhou expanded coverage. Lastly, we estimated utilization changes in aggregating the proportion of expenditures paid by the government health insurance fund for the study medicines. Future in-depth analyses are needed of who used these medicines, of who benefited from the insurance inclusion, and of population and individual health outcomes associated with expanded health insurance coverage.

## CONCLUSIONS

Before inclusion of the government health insurance program, expensive targeted anti-cancer medicines were not affordable even there are companies’ patient assistance programs. Government price negotiation and coverage inclusion reduced prices, stipulated utilization, and relieved patients’ financial burden to some extent. However, the burden is still heavy, especially for low income populations in rural areas. This calls for further price negotiations, careful monitoring and facilitation of appropriate use of the included expensive medicines, and of strategies to reduce patient financial burden to ensure appropriate care and a sustainable government health insurance system in China.
